# Electrical and Mechanical Performance of Carbon Fiber-Reinforced Polymer Used as the Impressed Current Anode Material

**DOI:** 10.3390/ma7085438

**Published:** 2014-07-24

**Authors:** Ji-Hua Zhu, Miaochang Zhu, Ningxu Han, Wei Liu, Feng Xing

**Affiliations:** Shenzhen Key Lab on Durability of Civil Engineering, College of Civil Engineering, Shenzhen University, Shenzhen 518060, China; E-Mails: szuzhumc@gmail.com (M.Z.); nxhan@szu.edu.cn (N.H.); liuwei@szu.edu.cn (W.L.)

**Keywords:** anode, anodic polarization, CFRP, current density, ICCP, steel-reinforced concrete

## Abstract

An investigation was performed by using carbon fiber-reinforced polymer (CFRP) as the anode material in the impressed current cathodic protection (ICCP) system of steel reinforced concrete structures. The service life and performance of CFRP were investigated in simulated ICCP systems with various configurations. Constant current densities were maintained during the tests. No significant degradation in electrical and mechanical properties was found for CFRP subjected to anodic polarization with the selected applied current densities. The service life of the CFRP-based ICCP system was discussed based on the practical reinforced concrete structure layout.

## 1. Introduction

Reinforced concrete structures may suffer from premature failure induced by the corrosion of the reinforcing steel embedded in the concrete, which implies that a huge investment in strengthening, repair and rehabilitation is needed in order to reach their targeted service lives [1]. It is well known that chloride ingress is one of the major causes of steel corrosion, which, in turn, leads to concrete cracking, due to the expansion exerted by corrosion products [2,3]. The chloride transport mechanism has been reported by Tang [4,5] and Li *et al.* [6]. The corrosion of steel in concrete is generally understood as an electrochemical phenomenon [7]. Nowadays, various well-developed methods are available for controlling steel corrosion in concrete [8]. One of the most effective methods, impressed current cathodic protection (ICCP) [9,10], can usually afford sufficient protection and, in some cases, has even been regarded as the only way to control steel corrosion [11]. In an ICCP system, a cathodic current is applied to the reinforcing steel, resulting in the shifting of the steel potential towards a level at which the corrosion rate is negligible [9,10].

It is of crucial importance to select a proper anode for the delivery of the protection current from the surface through the concrete to the steel rebar. Much research has been carried out by using different types of anodes, including thermal sprayed zinc anodes [12], thermal sprayed titanium anodes [13,14], titanium mesh [15] and conductive paint or overlay coating anodes [16,17]. The industry continually develops new anodes with requirements for bond efficiency, installation convenience and lower cost.

Carbon fiber-reinforced polymer (CFRP) consists of extremely strong and light carbon fibers embedded in a polymer matrix. CFRP is extensively used as a structural strengthening material, due to its sound mechanical properties, good durability and other considerations, such as aesthetics and ease of installation. Research on applications of CFRP in structures has been summarized by international specifications [18,19]. It should be noted that CFRP may also be a potential anode material in ICCP systems, due to its good electrical conductivity and electrochemical properties. For instance, it was shown that CFRP plates embedded in concrete may produce galvanic effects when coupled with steel bars [20].

There are relatively few publications with respect to the use of CFRP as the ICCP anode. Lee-Orantes *et al.* [21] presented an experimental investigation of using CFRP as anodes in ICCP of reinforced concrete prisms. The idea of using CFRP for both structural strengthening and ICCP of reinforced concrete structures was proposed. Gadve *et al.* [22,23] reported the test results of reinforced concrete lollipop specimens and beams with CFRP serving as an impressed current anode. Van Nguyen *et al.* [24] studied the performance of CFRP fabric and rod as impressed current anodes in calcium solution and concrete. In addition, CFRP was employed to pre-corroded reinforced concrete beams for both structural strengthening and ICCP, and the results showed that the ultimate strength of specimens with CFRP for dual functions (structural strengthening and ICCP) decreased slightly in comparison to control specimens only for structural strengthening [25,26].

The above publications were mainly focused on the corrosion behavior of steel reinforced concrete over application of ICCP with a CFRP anode. This paper, on the other hand, investigate the dual functional behaviors of CFRP, *i.e.*, electrical and mechanical behaviors, used as the impressed current anode material. The goals of the present research are: firstly, to investigate the influences of different solutions and current densities on the electrical and mechanical properties of CFRP plate by means of simulated ICCP systems; secondly, to establish an acceptable current density range for the CFRP plate used as an anode; and lastly, to further discuss the service life of a CFRP-based ICCP system.

## 2. Behavior of CFRP Plate in Simulated ICCP Systems

### 2.1. Specimen Preparation

**Figure 1:**
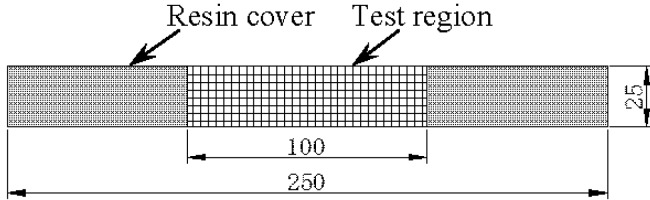
Geometric dimensions of the CFRP strip (mm).

Two groups of CFRP strips were prepared as shown in Table 1 and Table 2. Group 1 of 25 CFRP strips was tested to study the effect of long-term applied direct current, while Group 2 of 15 CFRP strips was for the corresponding effect in various solutions. The experimental program was designed by taking into considerations different possible functions of CFRP in an ICCP system, where CFRP plays the role of both a conductor and an electrode. The CFRP plate used is made of multi-layer carbon fibers bounded by LAM-125/LAM-226 laminating epoxy (Pro-Set Inc., Bay City, MI, USA). Each layer consists of weft-warp-knit carbon fibers combined by the epoxy. The carbon fiber used is Toray T700, and its volume fraction in CFRP is 60%. Table 3 gives information on the chemical composition of the epoxy in CFRP. The thickness of the CFRP plate is approximately 2 mm. The CFRP plates were cut into strips with the same geometries and dimensions, as shown in Figure 1. All strips were sand-blasted to remove superficial electrically nonconductive organics and to expose conductive parts of the CFRP strips. In addition, Group 2 of 15 CFRP strips was then coated with epoxy resin, except for the test region at the mid-length. The test region of each specimen was a rectangle of 100 mm in length and 25 mm in width, located in the center of a single side of the specimen, as shown in Figure 1. Therefore, the nominal anodic surface area (*A*_a_) of the specimen is 2500 mm^2^.

**Table 1:** Test series in Group 1.

Series	Current (mA)	Test duration (day)	No. of specimens
G1-RF	–	16	5
G1-I5	5	16	5
G1-I50	50	16	5
G1-I500	500	16	5
G1-I1000	1000	16	5

**Table 2:** Specimens of Group 2.

Series	Specimen	Current (mA)	Current Density (A/m^2^)	Solution
G2-RF	G2-RF-NaCl	–	–	NaCl
	G2-RF-Mix	–	–	Mix
	G2-RF-Ca(OH)_2_	–	–	Ca(OH)_2_
G2-i0.2	G2-i0.2-NaCl	0.5	0.2	NaCl
	G2-i0.2-Mix	0.5	0.2	Mix
	G2-i0.2-Ca(OH)_2_	0.5	0.2	Ca(OH)_2_
G2-i2	G2-i2-NaCl	5	2.0	NaCl
	G2-i2-Mix	5	2.0	Mix
	G2-i2-Ca(OH)_2_	5	2.0	Ca(OH)_2_
G2-i20	G2-i20-NaCl	50	20.0	NaCl
	G2-i20-Mix	50	20.0	Mix
	G2-i20-Ca(OH)_2_	50	20.0	Ca(OH)_2_
G2-i40	G2-i40-NaCl	100	40.0	NaCl
	G2-i40-Mix	100	40.0	Mix
	G2-i40-Ca(OH)_2_	100	40.0	Ca(OH)_2_

**Table 3:** Chemical composition of the polymer in CFRP.

Ingredient Name	Concentration (%)	Ingredient Name	Concentration (%)
Polyoxypropylenediamine	30–50	Aminoethylpiperazine	0–10
Formaldehyde, polymer with benzenamine, hydrogenated	10–20	1,3-Propandiamine, N,N'-1,2-ethandiylbis-	0–10
Cyclohexanediamine, 1,2-	10–20	Benzene-1,3-dimethanamine	0–10

### 2.2. Impressed Current Tests

The aim of the impressed current tests was to study the effect of direct current on the electrical and mechanical properties of CFRP plate. The 25 CFRP strips of Group 1 were categorized into five series according to the magnitude of the current flow to which they were subjected, as described in Table 1. The constant current densities of 0, 5, 50, 500 and 1000 mA were adopted, where 0 mA represented the reference specimens. The test specimens were labeled according to the applied current, as shown in Table 1. For example, the label “G1-I5” defines the specimen with the applied current of 5 mA. Each series included five parallel specimens, which were designated by a, b, c, d and e. The series of reference specimens were labelled as “G1-RF”, as shown in Table 1. During the impressed current test, each end of the CFRP strip was connected to either the terminal of a power supply, so as to constitute a closed circuit for current flow. Voltammetry was used to assess the electrical properties of each CFRP plate during the tests. The feeding voltage and current for each specimen were manually monitored and recorded every 12 h. Meanwhile, an infrared thermometer was used to monitor the temperature of the CFRP strips. The test period was 16 days.

**Figure 2:**
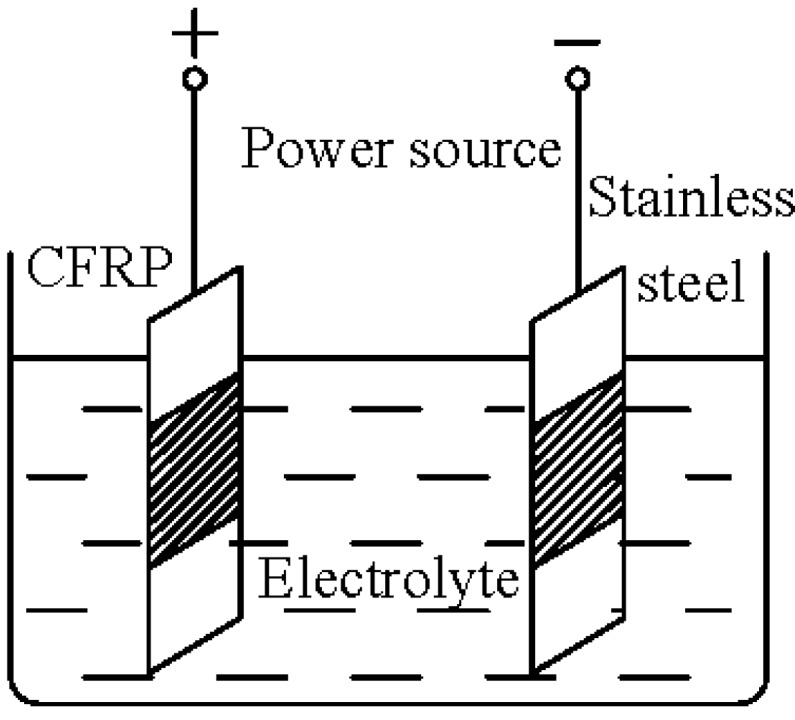
Schematic diagram of the test setup for simulated impressed current cathodic protection (ICCP) systems with CFRP.

In addition to direct current, CFRP plates as the anode in a real ICCP system would be subjected to anodic polarization. Therefore, it is also necessary to investigate their performance under realistic anodic polarization conditions. For the sake of simplicity, CFRP was employed as the anode material in simulated ICCP systems with different solutions, as shown in Figure 2. Anodic polarization was obtained by connecting CFRP strips (anode) and stainless steel strips (cathode) to the positive and negative terminals of a constant direct current source, respectively. The 15 CFRP strips of Group 2 were used to investigate the effect of anodic polarization on the CFRP plate in various solutions. Different solutions and current densities were adopted. Three kinds of solutions, namely, 3.5% NaCl solution (by mass percentage of solution), a mixture of saturated Ca(OH)_2_ solution with 1% NaCl (by mass percentage of solution) and saturated Ca(OH)_2_ solution, which were designated as NaCl, Mix and Ca(OH)_2_, respectively, were used as electrolytes in the simulated ICCP systems. The solutes used were of analytical grade, and the solvent was deionized water. The purpose of using different solutions was to investigate the CFRP plate’s resistance to chlorine and oxygen evolution in the different environments. According to the Faraday’s law, electrochemical reactions are strongly dependent on charge quantity. Thus, various current densities of 0.2, 2, 20 and 40 A/m^2^ were maintained during the tests. It should be noted that the current densities were calculated using the anodic surface area of each specimen.

The 15 specimens were separated into five series and labeled according to the applied current densities and solutions, as shown in Table 2. For example, the label “G2-i2-Mix” defines the specimen with the applied current density of 2 A/m^2^ and tested with the Mix solution. It should be noted that one series of specimens were tested in the three solutions without an applied current for reference purposes. This series of specimens were labelled “G2-RF”, as shown in Table 2.

The voltage between the CFRP and stainless steel strips was measured every 12 h for each specimen. In addition, the pH of the solutions was regularly monitored by using pH test strips with an accuracy of 0.5. It should be noted that no stirring and vibration were performed, so as to approximate the real working conditions of an anode as closely as possible. The test period was 25 days.

### 2.3. Tensile Test

**Figure 3:**
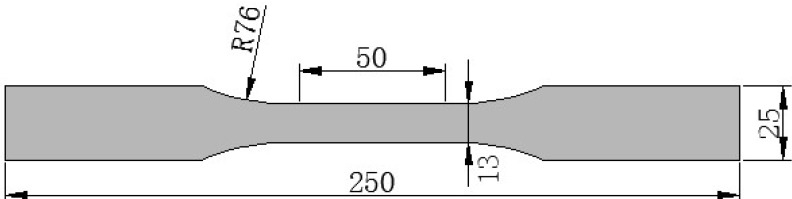
Geometric dimensions of the tensile coupon (mm).

Uniaxial tensile tests were carried out after the completion of the impressed current tests with both groups of CFRP strips. The intention of carrying out the tensile test was to evaluate the effects of current flow and anodic polarization on the mechanical properties of the CFRP plate. The CFRP strips were cut to be a dumb-bell shape, according to the ASTM Standard [27], as shown in Figure 3, in order to be sure that the tensile failure takes place in the targeted area. Strain gauges were attached in the middle of each specimen. The uniaxial tensile test was carried out with a universal test machine. A tensile loading rate of 0.2 mm/min was applied. The tensile force and strain of the CFRP strips were continuously monitored and recorded.

## 3. Test Results

### 3.1. Experimental Observations

The 25 CFRP strips of Group 1 subjected only to direct current did not show any significant changes of surface conditions. The temperature records also revealed no difference between the CFRP strips and the surrounding atmosphere during the test.

**Figure 4:**
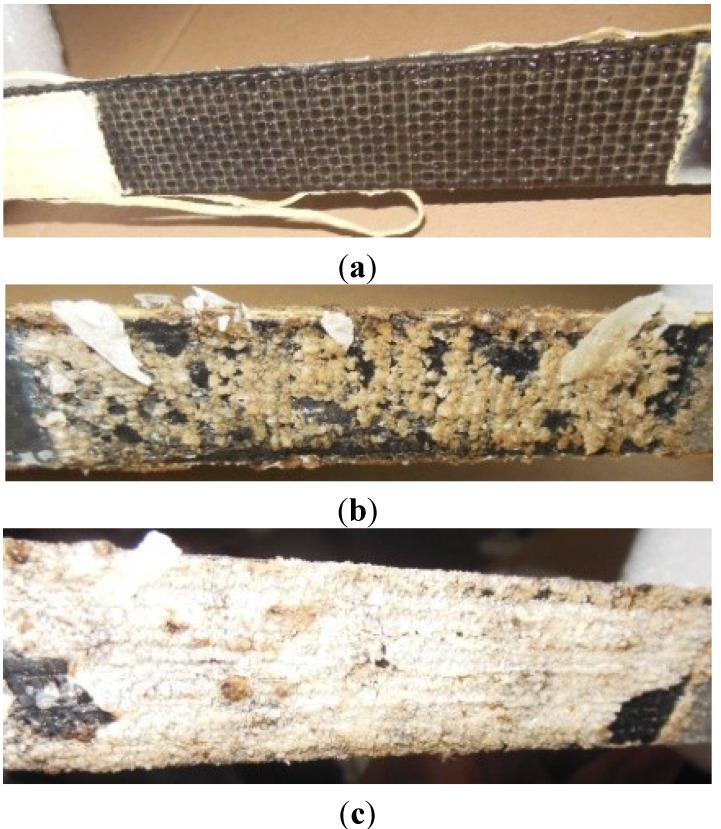
Surface conditions of Series G2I40 after 84 h of anodic polarization with a current density of 40 A/m^2^ in (**a**) 3.5% NaCl solution; (**b**) a mixture of saturated Ca(OH)_2_ solution with 1% NaCl; and (**c**) saturated Ca(OH)_2_ solution.

However, significant changes of surface conditions were observed in the CFRP strips of Group 2, which were subjected to the anodic polarization in various solutions. Some white and black sediment appeared on the CFRP strips in the saturated Ca(OH)_2_ solution and the Mix solution, while no sediment was observed for strips in the 3.5% NaCl solution. The amount of sediment increased with the applied current density. Figure 4 shows the surface conditions of CFRP strips subjected to a current density of 40 A/m^2^ after 84 h of anodic polarization in three different solutions. The underlying mechanism for CFRP’s degradation calls for further investigation. The other obvious change consisted of the thickness of the CFRP strips. The thickness increased to 100%–150% of the original value for strips subjected to current densities of 20 and 40 A/m^2^ in all three kinds of solutions. Significant swelling of CFRP was also found right at the border between the test area and the epoxy resin area for all specimens. No significant change of pH value of the testing solutions was observed during the tests.

### 3.2. Electrical Performance

**Figure 5:**
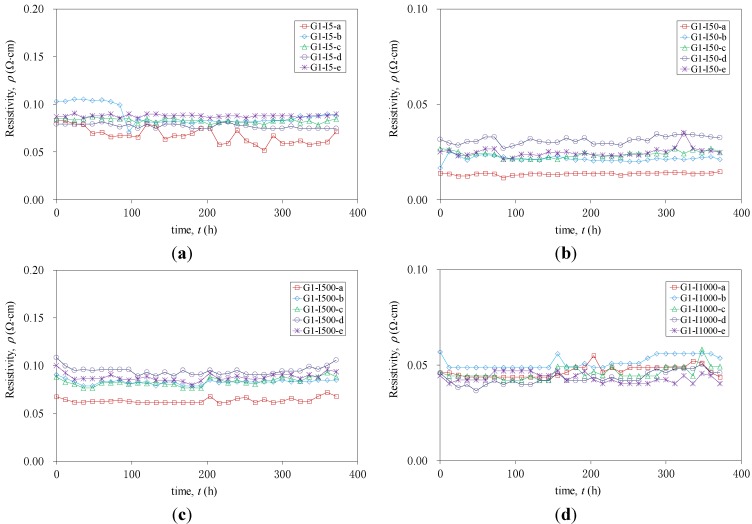
Resistivity *versus* time for each series of specimens in Group 1. (**a**) Series G1-I5; (**b**) Series G1-I50; (**c**) Series G1-I500; (**d**) Series G1-I1000.

There has been a lot of research on the mechanism of electrical conductivity in CFRP materials. It was recognized that factors, such as carbon fiber content, fiber arrangement and temperature, could have remarkable influences on the electric conductivity of CFRP materials [28,29,30]. The present work has focused on the effect of current flow on the electric conductivity of CFRP for a relatively long period. The electrical conductivity of the specimens was evaluated by resistivity over a test period. Once measuring the currents for a range of applied voltages, the resistances were calculated by using Ohm’s law. The resistivity could then be obtained using the resistance results and the geometric information of the specimens. Figure 5 plots the resistivity of CFRP strips (*R*) *versus* time (*t*) of Group 1, where the last label, a, b, c, d or e, refers to the five specimens of each series. It is clearly shown that the resistivity remained almost constant over test period. Although there was some variation of the resistivity between different series of specimens, values for all strips fell within the range of 0–0.1 Ω∙cm. The fluctuation could be attributed to material variations, environmental conditions and electrical connections.

**Figure 6:**
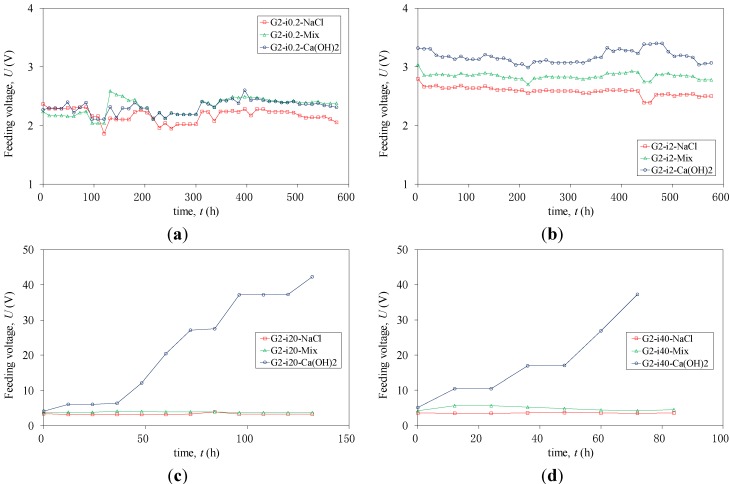
Measured feeding voltage *versus* time for each series of specimens in Group 2. (**a**) Series G2-i0.2; (**b**) Series G2-i2; (**c**) Series G2-i20; (**d**) Series G2-i40.

In the case of the 15 CFRP strips of Group 2 subjected to anodic polarization, analysis is complicated, due to the presence of several distinct sources of resistance in an ICCP system. These include solution resistance, stainless steel-solution interfacial resistance, CFRP-solution interfacial resistance and CFRP internal resistance. For simplicity, the conductive behavior was studied on the whole simulated ICCP systems by measuring the feeding voltage between CFRP (anode) and stainless strips (cathode). Figure 6 plots the feeding voltage (*U*) *versus* time (*t*) of Group 2 during anodic polarization. It is evident that *U* increased slightly with increasing applied current density. The measured voltage was the lowest for the 3.5% NaCl solution, followed by the Mix solution, and was the highest for the saturated Ca(OH)_2_ solution, as shown in Figure 6. Stable feeding voltages were observed for most of the tests, except for specimens G2I20-Ca(OH)_2_ and G2I40-Ca(OH)_2_. For these two specimens, *U* increased continuously with time, as shown in Figure 6c,d. The test was stopped when *U* reached around 40 V, due to the limitation of the power source capacity. The increased voltage was caused by an increasing accumulation of sediment on the surface of the CFRP strips, which could restrict the availability of the reactant. Consequently, a larger resistance appeared during anodic polarization.

### 3.3. Mechanical Properties

**Figure 7:**
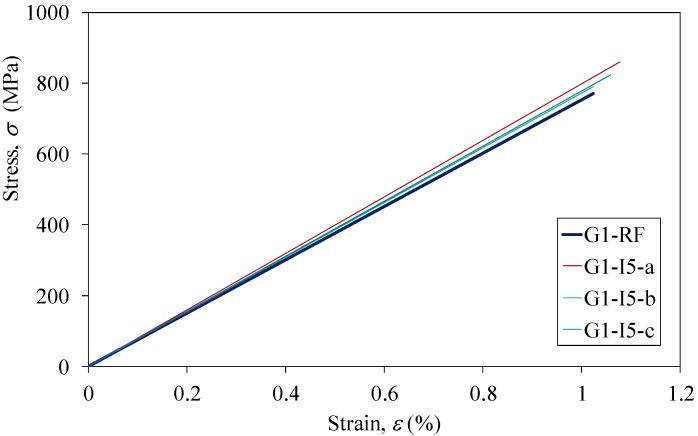
Measured stress-strain curves for reference specimen and Series G1-I5.

**Figure 8:**
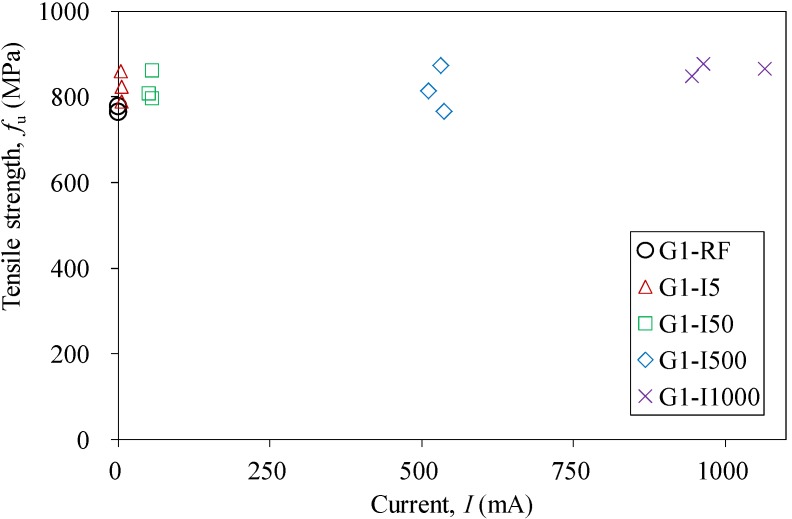
Comparison of tensile strength against current for specimens of Group 1.

**Figure 9:**
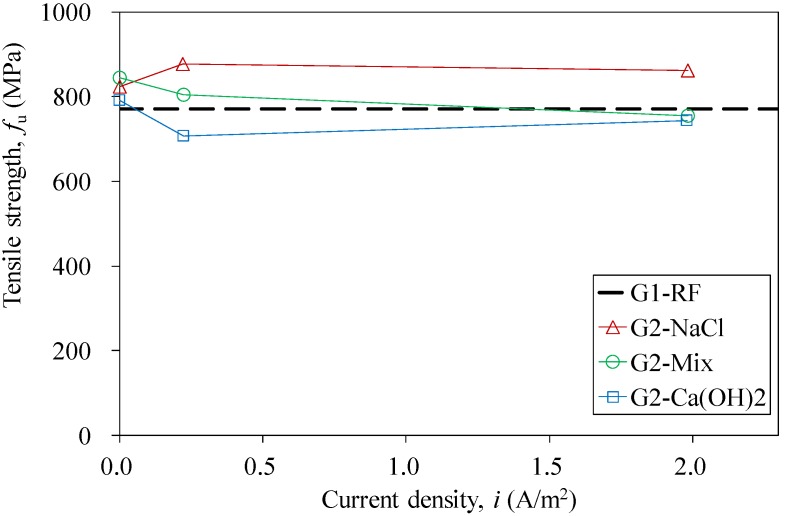
The relationship of tensile strength and current density for the reference specimen and different series specimens in Group 2.

Uniaxial tensile tests were carried out on both groups of the CFRP strips after the completion of the impressed current tests, except for the specimens of Series G2I20 and G2I40, since their excessive anodic polarization-induced swelling could nullify the mechanical properties. Almost all of the strips were found to have an identical tensile behavior, *i.e.*, the stress is proportional to the strain for each CFRP strip. Figure 7 shows the stress-strain curves of the specimens of G1RF (reference specimen) and Series G1I5. The mechanical properties obtained from tensile tests are summarized in Table 4, Table 5, Table 6 and Table 7. For the specimens of Group 1, the tensile strengths (*f*_u_) increased slightly with the increased applied current (*I*), whereas there was almost no change in elastic modulus (*E*_0_), as shown in Figure 8 and in Table 4, respectively. In contrast, both tensile strength and the elastic modulus decreased with increased applied current density for specimens of Group 2, subjected to anodic polarization in saturated the Ca(OH)_2_ solution and the Mix solution, as presented in Figure 9 and in Table 6 and Table 7. However, CFRP strips in 3.5% NaCl solution were found to have a small increase in tensile strength, as shown in Figure 9 and in Table 5.

**Table 4:** Measured mechanical properties for different series of specimens in Group 1.

Series	Test Results		Comparison	
	*f*_u_ (MPa)	*E*_0_ (GPa)	*f*_u_/*f*_u,RF_	*E*_0_/*E*_0,RF_
G1-RF	770.4	75.29	–	–
G1-I5	824.6	78.27	1.07	1.04
G1-I50	822.2	79.26	1.07	1.05
G1-I500	818.1	75.39	1.06	1.00
G1-I1000	863.8	75.78	1.12	1.01

Note: *f*_u_ = ultimate tensile strength; *f*_u,RF_ = ultimate tensile strength of the reference specimen; *E*_0_ = elastic modulus; *E*_0,RF_ = elastic modulus of the reference specimen.

**Table 5:** Measured mechanical properties for specimens of Group 2 tested with the 3.5% NaCl solution.

Specimen	Test Results		Comparison	
	*f*_u_ (MPa)	*E*_0_ (GPa)	*f*_u_/*f*_u,RF-NaCl_	*E*_0_/*E*_0,RF-NaCl_
G2-RF-NaCl	824.0	76.24	–	–
G2-i0.2-NaCl	877.0	73.93	1.06	0.97
G2-i2-NaCl	861.2	73.78	1.05	0.97

Note: *f*_u,RF-NaCl_ = ultimate tensile strength of the reference specimen with the 3.5% NaCl solution; *E*_0,RF-NaCl_ = elastic modulus of the reference specimen with the 3.5% NaCl solution.

**Table 6:** Measured mechanical properties for specimens in Group 2 tested with the Mix solution.

Specimen	Test Results		Comparison	
	*f*_u_ (MPa)	*E*_0_ (GPa)	*f*_u_/*f*_u,RF-Mix_	*E*_0_/*E*_0,RF-Mix_
G2-RF-Mix	843.8	77.42	–	–
G2-i0.2-Mix	804.9	73.18	0.95	0.95
G2-i2-Mix	754.3	74.66	0.89	0.96

Note: *f*_u,RF-mix_ = ultimate tensile strength of the reference specimen with the Mix solution; *E*_0,RF-mix_ = elastic modulus of the reference specimen with the Mix solution.

**Table 7:** Measured mechanical properties for specimens in Group 2 tested with the saturated Ca(OH)_2_ solution.

Specimen	Test Results		Comparison	
	*f*_u_ (MPa)	*E*_0_ (GPa)	*f*_u_/*f*_u,RF-Ca(OH)2_	*E*_0_/*E*_0,RF-Ca(OH)2_
G2-RF-Ca(OH)_2_	790.3	73.46	–	–
G2-i0.2-Ca(OH)_2_	707.1	68.38	0.89	0.93
G2-i2-Ca(OH)_2_	743.4	66.38	0.94	0.90

Note: *f*_u,RF-Ca(OH)2_ = ultimate tensile strength of the reference specimen with the saturated limewater; *E*_0,RF-Ca(OH)2_ = elastic modulus of the reference specimen with the saturated Ca(OH)_2_ solution.

## 4. Discussion of Service Life

The service life of an impressed current anode characterized by its capacity to transfer charge through the anode/electrolyte interface can be evaluated according to the NACE specification [31]. The capacity to transfer charge is defined as the total charge quantity (*Q*_anode_) passed by the anode during the application of ICCP, which can be calculated by Equation (1).

*Q*_anode_ = *i*_a_ × *t*_g_ × *A*_a_ (1)

where: *i*_a_ = applied anodic current density; *t*_g_ = duration of impressed current; *A*_a_ = anodic surface area.

Based on the above-mentioned discussion, the current density of 2 A/m^2^ with a duration of impressed current of 25 days could be considered as a conservative assessment of the service life of the CFRP plate used as an anode in the simulated ICCP systems without significant degradation of both the electrical and mechanical properties. Therefore, the capacity of the CFRP plate to transfer charge (*Q*_CFRP_) can be calculated according to Equation (2).

*Q*_CFRP_ = *Q*_anode_ = *i*_a_ × *t*_g_ × *A*_a_ = 4.32 × 10^6^*A*_a_ (*C*) (2)

It is no doubt that the service life of a practical ICCP system is dependent not only on *Q*_anode_, but also on the steel reinforcement configuration and other factors, such as the anode/concrete interfacial properties and concrete quality. This paper focuses on the behavior of CFRP in the ICCP system. Therefore, by assuming that *Q*_anode_ is the governing factor, it is possible to evaluate the service life of an ICCP system based on the equilibrium of charge quantity between the cathode *(Q*_cathode_) and anode (*Q*_anode_), as shown in Equation (3).

*Q*_cathode_ = *Q*_anode_ (3)

**Figure 10:**
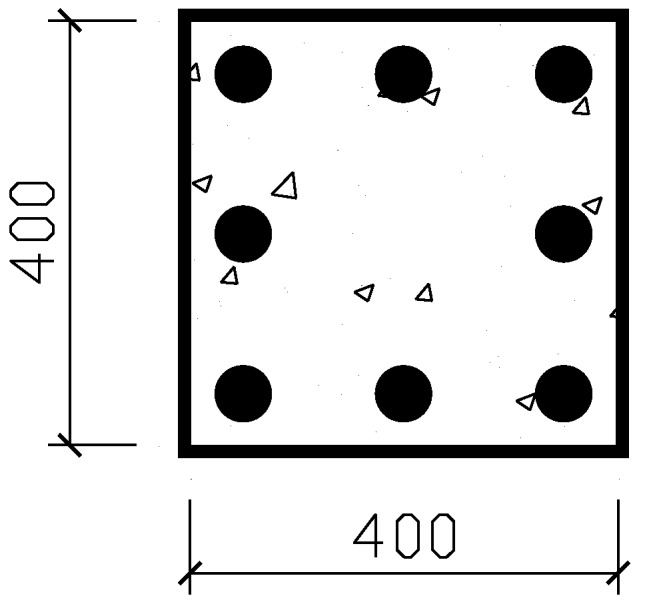
An eight-steel rebar-reinforced concrete element wrapped with CFRP plate as an anode.

The service life was investigated by using a typical concrete cross-section, as shown in Figure 10. A concrete element with a cross-section of 400 × 400 mm^2^ was reinforced by eight identical steel rebars. The ICCP was applied by wrapping a CFRP plate around the concrete element, where the CFRP plate and the steel rebar serve as the anode and the cathode, respectively. It was assumed that each steel rebar receives the identical protection current density (*i*_p_) throughout protection, and Equations (1)–(3) were adopted. The unit length of the concrete element was considered. Therefore, the charge quantity of steel (*Q*_steel_) could be calculated by Equation (4).

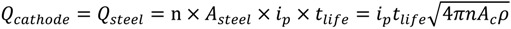
(4)
where: *n* = number of steel rebars; *A*_steel_ = steel surface area of unit length in contact with concrete; *i*_p_ = applied protection current density of cathode (steel); *A*_c_ = cross-sectional area of concrete element; ρ = reinforcement ratio of concrete element, which can be calculated by dividing the cross-sectional area of concrete by the total cross-sectional area of steel rebars in concrete; *t*_life_ = service life of an ICCP system governed by *Q*_CFRP_.

Hence, *t*_life_ can be calculated by inserting Equation (4) into Equation (3). The protection current density of 2–20 mA/m^2^ is recommended for ICCP of corrosion-deteriorated-reinforced concrete structures [32]. Two protection current densities of 5 and 20 mA/m^2^ and varied reinforcement ratios from 0.6% to 5%, as commonly used in practice as the lower and the upper limits, were used in the calculation.

**Figure 11:**
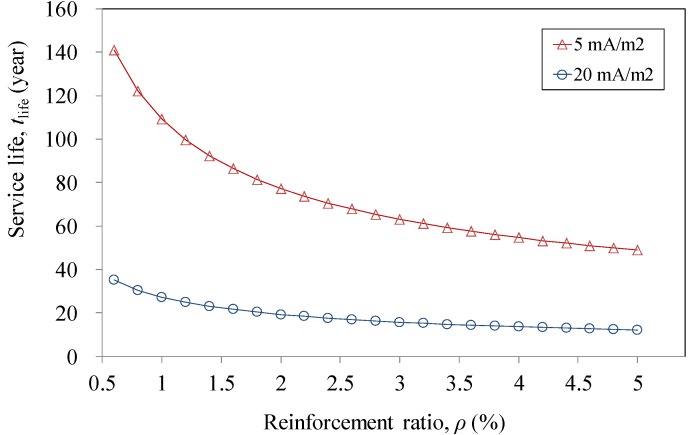
Comparison of predicted service life against reinforcement ratio for different current densities in ICCP of reinforced concrete.

In Figure 11, the predicted service life (*t*_life_) *versus* reinforcement ratio (ρ) of two adopted protection current densities (*i*_p_) is compared. It is shown that *t*_life_ decreases with increased reinforcement ratio. When the reinforcement ratio reaches the lower limit of 0.6%, the service lives are 140 and 35 years, with the corresponding protection current densities of 5 and 20 mA/m^2^, respectively. When the reinforcement ratio reaches the upper limit of 5%, the service lives are 50 and 12 years, with the corresponding protection current densities of 5 and 20 mA/m^2^, respectively. It is demonstrated that the CFRP plate can be successfully used as the anode material in the ICCP system over an acceptable service period, without significant degradation of the electrical and mechanical properties of CFRP. However, it should be noted that the above-mentioned discussion was obtained without considering the behavior of the anode/concrete interface, which is necessary for further investigations.

## 5. Conclusions

A comprehensive experimental program was carried out to study the electrical and mechanical behaviors of a CFRP plate in simulated ICCP systems with varied solutions. The following conclusions can be drawn based on the current research:

(1) Stable electrical and mechanical behaviors were observed in the experiments operated with only direct current.
(2) Tests were also carried out in simulated ICCP systems with various solutions. No significant degradation in both electrical and mechanical performances was found for CFRP strips operated with current densities of 0.2 and 2 A/m^2^.
(3) It is demonstrated that the CFRP plate can serve as the anode material in the ICCP system. The minimum predicted service life is 12 years, even with the maximum acceptable protection current density and reinforcement ratio. It should be noted that the prediction is conservative.

## Notation

*A*_a_: Anodic surface area of CFRP
*A*_c_: Cross-sectional area of the concrete element
*A*_steel_: Surface area of each steel rebar in the concrete element
*E*: Steel potential measured against CSE
*E*_0_: Elastic modulus for CFRP strips
*E*_0,RF_, *E*_0,RF-NaCl_, *E*_0,RF-Mix_, *E*_0,RF-Ca(OH)2_: Elastic modulus for G1RF, G2RF-NaCl, G2RF-Mix, G2RF-Ca(OH)_2_, respectively
*f*_u_: Ultimate tensile strength of CFRP strips
*f*_u,RF_, *f*_u,RF-NaCl_, *f*_u,RF-Mix_, *f*_u,RF-Ca(OH)2_: Ultimate tensile strength for G1RF, G2RF-NaCl, G2RF-Mix, G2RF-Ca(OH)_2_, respectively
*I*: Current
*i*: Current density
*i*_p_: Protection current density
*n*: Number of steel rebars in the concrete element
*Q*_anode_: Anode’s capacity to transfer charge
*Q*_cathode_: Total charge quantity passed the cathode during cathodic protection
*Q*_CFRP_: CFRP plate’s capacity to transfer charge
*Q*_steel_: Total charge quantity passed the steel in the concrete element during cathodic protection
*R*: Resistance
*t*: Time
*t*_g_: Duration of impressed current
*t*_life_: Predicted service life of the ICCP system by QCFRP
*U*: Voltage
ρ: Reinforcement ratio
σ: Tensile stress
ε: Tensile strain
